# A Costly Experience

**Published:** 1896-11

**Authors:** 


### A COSTLY EXPERIENCE.

"WHEN one door opens, another closes," and the lessons to be learned should be fruitful of good to all. The value of governmental aid to the school in the Empire State will now be tested, and much is expected of it in the solving of higher veterinary education for our associates and successors. The dangers of private undertakings and one-man institutions have long been a source of deep concern and earnest thought for those who have striven at all times to advance veterinary science in America. Another page is added to this history by the closing of the doors at Cincinnati; and what is the cost? What are the results? Some will say not serious in extent; which perhaps is true; but how serious in character only those can appreciate who have labored in the educational field, and will be bitterly regretted by those who entered upon the undertaking as a money-making venture—a stock company where great returns were looked for from the investment, and who are now vexed and disturbed by lawsuits and referee hearings in court, whereby they may escape from as much costly experience as the technicalities of the law will allow; some of the stockholders insolvent, who perhaps never invested a dollar; a number of young men throughout the country without parental oversight, so defectively educated that the strong arms of the national association cannot receive them, and perhaps barred out in the future from some of the State organizations. In disgrace its first leader was forced from its directorship, and the many changes that followed seemed only to hasten the inevitable end; a number of promising young veterinarians drawn to its staff of instructors, hoping thereby to attain the honors that would naturally follow in the train of their vocation, are now drinking the cup of sorrow, as they realize what a glittering bauble the whole affair was, and how empty an honor when it comes freighted with conditions that were essential, first, last, and all the time, for the perpetuation of schools started under such auspices. Born of no scientific parental love, devoted to no higher aspects of educational advancement than a business undertaking could create, it dies unlamented and will soon be forgotten, with the hope that it will never be resuscitated under such auspices.

